# Acutely Obstructed Airway Resulting from Complications of a Laryngopyocoele

**DOI:** 10.1155/2017/8123573

**Published:** 2017-05-18

**Authors:** Rosalind Mole, Stephen Hayes, Simon Dennis

**Affiliations:** Salisbury NHS Foundation Trust, Salisbury SP2 8BJ, UK

## Abstract

Laryngocoeles are rare cystic dilatations of the laryngeal ventricle. Obstruction of its outlet can cause entrapment of mucus and superimposed infection causes a laryngopyocoele. Such presentations, although rare, have potential to cause airway obstruction. A 67-year-old lady presented with a one-week history of hoarseness and shortness of breath. On examination, she was stridulous and had fullness of the left side of the neck. Nasendoscopy revealed large bilateral vocal cord polyps and near-complete glottis obstruction. She was taken to emergency theatre for restoration of a viable airway. Upon excision of the polyps, pus was visualised originating from the laryngeal ventricle. Literature proposes that laryngocoeles develop secondary to a one-way valve caused by an obstructing lesion distorting the saccule neck. We propose that the laryngocoele developed secondary to large obstructing polyps. Urgent excision of the polyps allowed decompression of the laryngopyocoele and reestablishment of a patent airway.

## 1. Introduction

Laryngocoeles are a rare dilatation of the laryngeal ventricle which can present in a variety of ways ranging from benign neck lumps to acute airway obstruction. The aetiology of laryngocoeles remains unclear, but a number of associations have been described. We present a case of a 67-year-old lady who presented with near-complete glottis obstruction. Initial examination revealed severe vocal cord polyps and she underwent emergency debridement. Debridement of the polyps resulted in the discovery of a pus-filled laryngocoele. This case represents the first description in the literature of coexisting vocal cord polyps and laryngocoeles. By considering previously described theories on the development of laryngocoeles, we speculate that the severity of the polyps in this case resulted in insufficient drainage of the laryngeal ventricle and the development of the laryngocoele.

## 2. Case History

**Figure 1:**
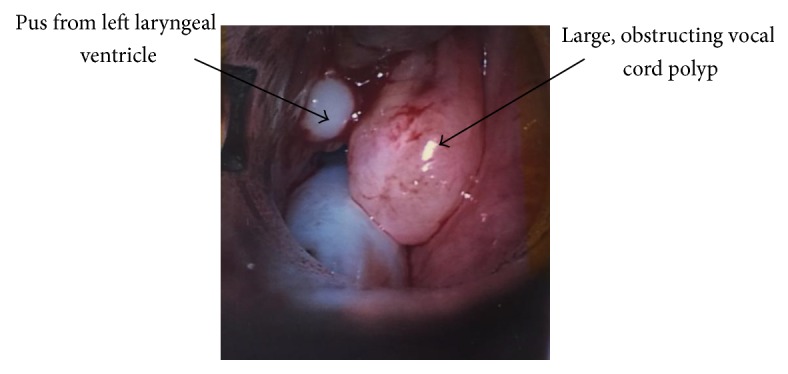
Intraoperative image demonstrating pus from left laryngeal ventricle.

A 67-year-old lady presented to the Ear, Nose and Throat emergency clinic with a one-week history of hoarseness and intermittent shortness of breath, worse at night. She had a 40-pack-year history and was fearful of seeking medical attention. On examination, she was afebrile, stridulous at rest, tachypnoeic, tachycardic, and hypertensive. On palpation, there was fullness to the left side of her neck. Flexible laryngoscopy revealed large bilateral vocal cord polyps causing almost complete airway obstruction. The findings of such extensive polyps on laryngoscopy implied a potentially longer clinical course than originally reported. The patient underwent an emergency microlaryngoscopy under general anaesthetic for debridement of the vocal cord polyps in order to establish a definitive airway. On excising the left vocal cord polyp, pus appeared to be discharging from the left laryngeal ventricle (Figure 1). Drainage of the ventricle was aided with external pressure. Swabs were taken.

Due to extensive airway oedema and the risk of recollection and aspiration, the decision was made for the patient to remain intubated on ITU postoperatively. Postoperative CT scan of the patient's neck demonstrated no recollection and the patient was extubated 2 days later and was discharged after 1 week. Intraoperative pus swabs isolated* Haemophilus influenzae* and* Beta-haemolytic Streptococcus* and histology was consistent with vocal cord polyps with no indication of malignancy.

At follow-up appointments at 8 weeks and 3 months, the patient was well with no further airway compromise. Flexible laryngoscopy revealed healthy vocal cords with no evidence of recollection within the ventricle.

## 3. Discussion

Laryngocoeles are herniations of the laryngeal ventricle [1]. They are rare with an estimated incidence of 1 per 2.5 million people per year [2]. Laryngocoeles are typically air-filled but should the neck of the ventricle become obstructed mucus can collect within the ventricle and create a laryngomucocoele. Infection of the mucus within the ventricle leads to the development of a laryngopyocoele. This is a very rare presentation. Approximately 8% of laryngocoeles present as laryngopyocoeles. There have been fewer than 50 cases described in the literature worldwide [1]. Within this case report, the laryngopyocoele was identified intraoperatively after pus was observed discharging from the ventricle. This was confirmed microbiologically, isolating* Haemophilus influenzae* and* Beta-haemolytic Streptococcus*.

In this case, the laryngopyocoele was drained intraoperatively with external compression. It was irrigated with normal saline. There was no recurrence at follow-up using this technique. Endoscopic marsupialisation using a CO_2_ laser has been described in the literature as an option for treatment of laryngocoeles to prevent recurrence. An alternative treatment was described by Fraser et al. This paper presented a similar case as described here and discussed endoscopic marsupialisation of the laryngopyocoele using a microdebrider [3]. This is a novel technique making use of readily available theatre equipment. This is a technique that should be considered for larger laryngocoeles in order to reduce the chance of recurrence.

The aetiology of laryngocoeles remains unknown, but some associations have been reported within the literature. One of the most well described associations includes raised intraglottic pressure, which in itself can have a number of different aetiologies. For example, wind or brass musicians have a higher incidence of laryngocoeles compared to the rest of the population [4, 5]. There have also been a number of conditions considered to be associated with laryngocoeles. Broadly, these can be classified into intralaryngeal (including carcinoma, amyloidosis, and papillomatosis) and extralaryngeal (including ankylosing spondylitis, rheumatoid arthritis, and oncocytic cysts) [2, 4, 5].

There have been many studies making a connection between laryngeal carcinoma and laryngocoeles [5]. The coexistence of the conditions was first described by Marschik in 1927. Since then the incidence of laryngocoeles in laryngeal carcinoma has been quoted anywhere between 0.16 and 18%. The broad range is likely to be due to the variation in measurement techniques and diagnostic criteria [5]. Several theories have been postulated regarding the mechanism behind the association. One theory is that of a one-way valve, where the carcinoma distorts the neck of the ventricle causing it to inflate. An alternative theory is that the presence of the carcinoma alters the physiology of the larynx and increases the pressure within the lumen through a combination of factors, for example, frequent coughing, phonatuary misuse, or changes in neuromuscular mechanics [5].

To our knowledge, this case represents the first report of a laryngocoele coexisting with vocal cord polyps. We hypothesise that increased intralaryngeal pressure was caused by the vocal cord polyps obstructing the neck of the laryngeal ventricle leading to laryngocoele formation, in a very similar fashion to laryngeal carcinoma. The vocal cord polyps in this case were very extensive due to the patient being fearful of seeking medical attention. Therefore the unusually long length of the clinical course of this common presentation is likely to have been a contributing factor towards the development of such an uncommon condition: a laryngopyocoele.

The development of a laryngocoele or laryngopyocoele is extremely rare but may develop in coexistence with more common pathology, such as vocal cord polyps. We present this case to highlight severe vocal cord polyposis as a potential association with laryngocoele formation.
